# The complete chloroplast genome of *Suaeda physophora* Pall. (Chenopodiaceae)

**DOI:** 10.1080/23802359.2022.2115322

**Published:** 2022-09-02

**Authors:** Yanlin Hu, Xueli Ren, Jiayin Zhang

**Affiliations:** a Gansu Sanrui Agritec Co., Ltd., Gansu, China; b School of Life Sciences, Ministry of Education Key Laboratory for Biodiversity Science and Ecological Engineering, Institute of Biodiversity Science, Fudan University, Shanghai, China

**Keywords:** *Suaeda physophora*, chloroplast, phylogeny

## Abstract

*Suaeda physophora* Pall. (Chenopodiaceae) is a leaf succulent shrub species with potential usefulness as fodder for the desert animal. However, the phylogeny of *S. physophora* is lacking. Here, we sequenced and assembled a complete chloroplast genome of *S. physophora* and further reconstructed the phylogeny of Chenopodiaceae. The chloroplast genome of *S. physophora* is 151,104 bp in length, consisting of an 18,597 bp small single-copy (SSC), an 82,845 bp large single-copy (LSC), and a pair of 24,831 bp inverted repeat (IR) regions. The genome encodes 131 genes, including 87 protein-coding genes, 36 tRNA genes, and eight rRNA genes. Phylogenetic analysis revealed that the genus *Suaeda* forms a monophyletic taxon, and *S. physophora* is closely related to *S. eltonica*. Chloroplast genome and phylogenetic studies provided an essential foundation for the conservation of *S. physophora*.

*Suaeda physophora* Pall. (1803) is a salt-resistant halophyte distributed in northwestern China, Eastern Europe, Central Asia, and Western Siberia (Song et al. 2005). Owing to its importance as fodder in areas with saline soil, the salt-tolerance mechanisms of *S. physophora* have been extensively studied (Song et al. 2006; Li and Zhang 2007; Yuan et al. 2010; Yang et al. 2016). However, the phylogeny of this species is not comprehensively resolved. A previous study used one nuclear locus, two plastid loci, and morphological characteristics to infer the phylogeny and taxonomy of Chenopodiaceae (Schütze et al. 2003); as only *atp*B-*rbc*L reference sequences were available, *S. physophora* was grouped together with *S. palaestina* and *S. ifniensis*; however, their phylogenetic positions were not yet resolved. Thus, more comprehensive and stronger molecular evidence is still needed, especially at the plastomic level. To address this, we assembled and decoded the chloroplast genome of *S. physophora*, representing the first complete chloroplast genome of this species.

We collected samples of *S. physophora* growing wild in Xinjiang, China (42.8488° N, 89.1975° E), in accordance with local laws and exerting no environmental damage. Species identification was based on morphology and subsequent chloroplast genome sequencing. The *S. physophora* specimen was deposited at the Molecular Evolution and Ecology Laboratory, Fudan University (contact: Jiayin Zhang, email: jiayinzhang16@fudan.edu.cn), voucher Sa004-HP002-Sphy. Total genomic DNA was extracted using Plant DNAzol Reagent (Thermo Fisher Scientific, Waltham, MA) according to the manufacturer`s instructions and was sequenced on an Illumina Hiseq 2500 platform (Illumina, San Diego, CA) by a commercial service provider (BerryGenomics, Beijing, China) in 150-bp paired-end sequencing mode. The *S. physophora* chloroplast genome was assembled using GetOrganelle v1.7.5 (Jin et al. 2020), followed by manual correction using Bandage v0.8.1 (Wick et al. 2015). The final assembly was curated using Pilon v1.22 (Walker et al. 2014), and annotation was performed on GeSeq (Tillich et al. 2017) with all available chloroplast genomes of *Suaeda* (Chenopodiaceae) as references. The assembled genome sequence and annotations were submitted to GenBank (accession no. ON571659).

The assembled *S. physophora* chloroplast genome was 151,104 bp long, including an 18,597-bp small single-copy (SSC), an 82,845-bp large single-copy (LSC), and a pair of 24,831-bp inverted repeat (IR) regions. We annotated 131 genes, including 87 protein-coding genes, eight rRNA genes, and 36 tRNA genes, among which 10 tRNA genes, four rRNA genes, and eight protein-coding genes were duplicate in IR regions. The average GC content of the LSC, SSC, and IR regions is 34.3%, 29.0%, and 42.8%, respectively.

To resolve the taxonomic status at plastomic level, the complete chloroplast genomes of *S. physophora* and 38 other species belonging to the Chenopodiaceae and Amaranthaceae were used to construct a phylogenetic tree. MAFFT v7.487 (Rozewicki et al. 2019) and TrimAl v1.4.rev22 (Capella-Gutiérrez et al. 2009) were used to align and trim the genome sequences. We used IQ-TREE v2.1.2 (Minh et al. 2020) to construct a maximum-likelihood tree with a TVM + F+R4 model. Phylogenetic analysis results (Figure 1) strongly suggested that the genus *Suaeda* is monophyletic, and *S. physophora* is a sister species to *S. eltonica*.

**Figure 1.**
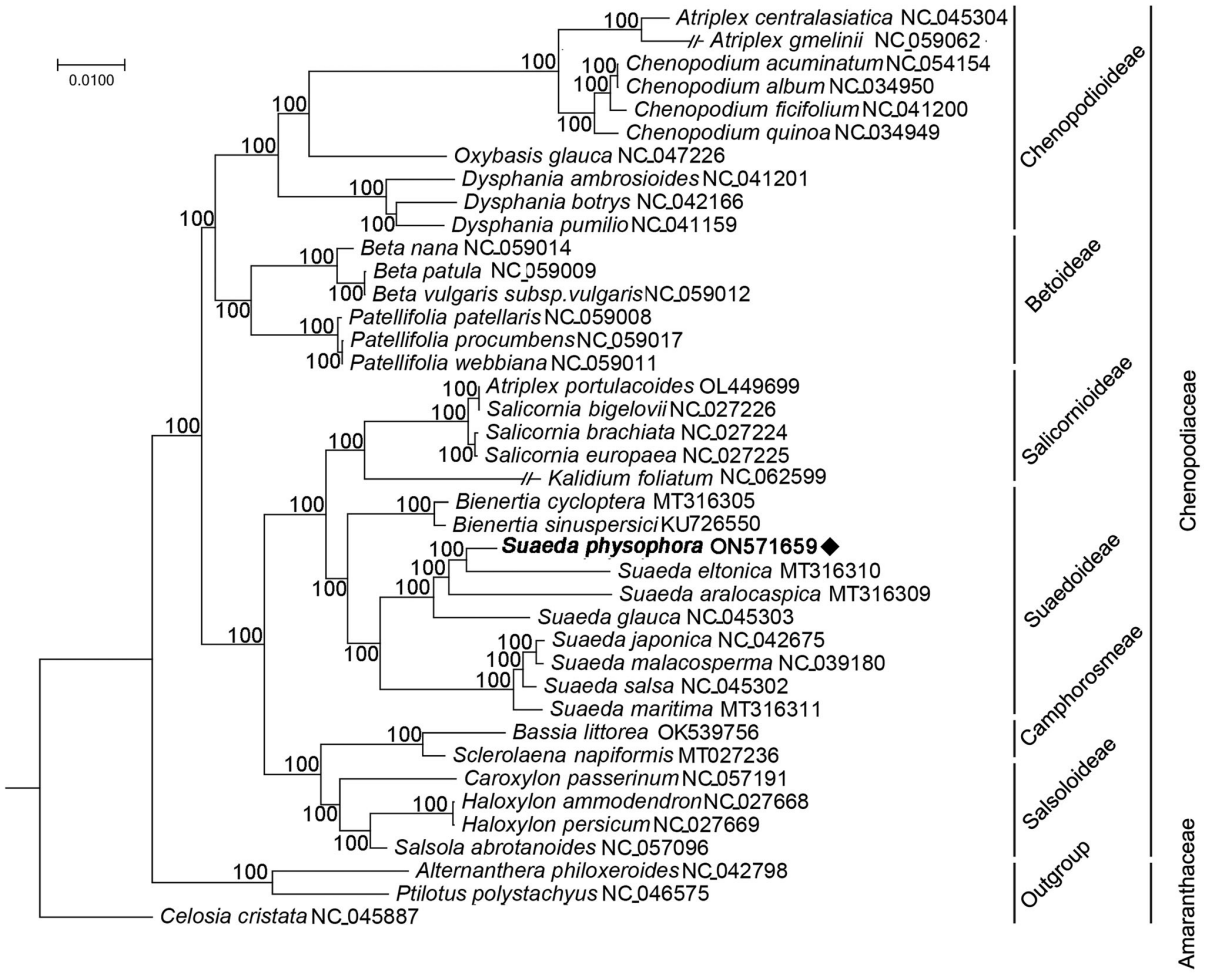
A phylogeny tree of Chenopodiaceae based on complete chloroplast genomes with three Amaranthaceae species as outgroup. The maximum-likelihood (ML) tree was generated with 3000 bootstrap replicates. The target plant was marked with ♦.

## Data Availability

The genome sequence data that support the findings of this study are openly available in GenBank of NCBI at https://www.ncbi.nlm.nih.gov under the accession no. ON571659. The associated BioProject, SRA, and Bio-Sample numbers are PRJNA844225, SRR19501374, and SAMN28798005, respectively.
